# The Effect of Indian Fig Fruit Extract on Human Papilloma Virus containing Cervical Cancer Cells (HeLa) by Decreasing the *HPV18 L1* Gene Load

**DOI:** 10.31557/APJCP.2021.22.3.785

**Published:** 2021-03

**Authors:** V M Berlin Grace, Lydia B, D David Wilson

**Affiliations:** 1 *Department of Biotechnology & Health Sciences, Karunya Institute of Technology and Sciences, Karunya Nagar, Coimbatore-641 114, Tamil Nadu, India. *; 2 *School of Science, Arts, Media and Management (SSAMM), Karunya Institute of Technology and Sciences, Karunya Nagar, Coimbatore-641 114, Tamil Nadu, India. *

**Keywords:** HeLa cells, HPV18, viral load, PCR, cytotoxicity, DNA fragmentation

## Abstract

**Background:**

Global trend is moving towards the use of natural phytochemicals to fight against pathogens. Human cervical cancer is directly associated with onco-potent type of Human Papilloma Virus (HPV). There is no known medicine for clearance of HPV type whose persistence is the cause of occurrence and re-occurrence of cervical cancer. The different species of fig fruit and their latex are reported to have HPV associated genital warts clearance capability.

**Methods:**

In the current investigation, the effect of the methanol extract of Ficus benghalensis L. fruits on HPV type18 viral load in HeLa cell line was tested by doing PCR using HPV L1 primers (MY09/My011) and the cytotoxicity was also analysed by MTT assay. The induction of apoptotic activity in terms of DNA fragmentation and hyper-chromic effects of DNA was analysed.

**Results:**

The PCR results showed a reduction in the HPV18 DNA and also the treatment exhibited a promising cytotoxicity with IC50 value at 211.86 μg/ml. The DNA samples from treated HeLa cells showed DNA shearing and laddering as a mark of apoptotic DNA fragmentation (Fig. 2) and the UV absorbance value at 260 nm was found to be significantly (P<0.01) higher in the DNA sample treated with fruit extract compared to the untreated DNA sample.

**Conclusion:**

The Ficus benghalensis L. fruit extract reduced the HPV viral load in HPV18 containing HeLa cells and showed an effective cytotoxicity on HeLa cell line. It also could induce the apoptotic activity in HeLa cell line and this study results suggest that the Ficus benghalensis L. fruits can be used to fight against cervical carcinoma, acting on HPV load.

## Introduction

Cancer is a life threatening disease, which is not having any particular cure at present. Cervical cancer is the second most common types of cancer in women worldwide. In the current scenario, the incidence and mortality of cervical cancer is less due to the implementation of advanced prevention and screening techniques. Human Papilloma Virus (HPV) is the major causative agent for the development of cervical cancer as it is principally transmitted by sexual mode. The HPV is a group of over 200 different related types, in which about 40 HPV serotypes infecting genital tract cause various lesions (Eileen, 2003). Among the HPV types, few are identified as onco-potent types, which cause approximately 5% cancer burden of all cancers worldwide and are categorized as high risk types (De Martel et al., 2012). The high and low risk HPV types are mainly transmitted sexually before the symptoms appear on the infected person and it also can be transmitted non-sexually by birth or via mothers milk or by close contact as per the data of last 2 decades (Sabeena et al., 2017). More than 90% of cervical cancer and few non cervical cancers like anal, vulvar and oesophageal are caused by high risk type HPV 16/18 (Lowry and Schiller, 2012). According to the IARC report, the cause of several cancers of oropharynx, base of the tongue, tonsils, cervix uteri, penis, vulva, vagina and anus origin have shown a strong relationship with HPV infection (Dania et al., 2016).

HPV is a non-enveloped, epitheliotrophic virus with double stranded circular DNA of 7900 bp with eight overlapping open reading frames (ORF). The HPV 18 genome is of 7837 bp with 6 early (E) genes (E1, E2, E4, E5, E6 and E7), which code for non-structural regulatory proteins for the replication and other transformation related functions of HPV (Ramírez-Fort et al., 2014). The late (L) genes, L1 and L2 encode the major and minor capsid proteins (the capsid contains 72 pentamers of L1 gene and 12 molecules of L2 gene), which are responsible in viral particle formation for subsequent infection to other cells. The early genes regulate viral replication whereas; a few of them have the transformation potential, which makes the treatment difficult (Molijn et al, 2005). The HPV type, viral load and integration status of HPV predict the patient’s prognosis (Walboomers et al., 1999; Josefsson et al., 2000). Several molecular testing devices such as; signal amplification methods (Cervista^®^ HPV HR) and nucleic acid amplification methods (Microarray, Polymerase Chain Reaction (PCR), PapilloCheck^®^) have been approved by FDP for screening high risk type HPV infection in addition to the cytological Pap test. The detection of HPV by polymerase chain reaction (PCR) is the main FDA approved method for screening high risk type HPV in clinical samples due to its high sensitivity and reliability (Heidi et al., 1991; Andre et al., 2012). All the diagnostic methods have reported a high prevalence of high risk type HPV16/18 in cervical cancer. We have also reported 95% invasive cervical cancer cases infected with onco-potent HPV types HPV16/18 using PCR based detection method (Grace et al., 2006). As the persistence of onco-potent HPV in epithelial cells is responsible for the development and recurrence of cervical cancer, the viral load needs to be eliminated in all the HPV infected cells. The HeLa cell line is isolated from adenocarcinoma of uterine cervix and it contains HPV type 18 as viral particles as well as an integrated form as per many published research articles (Badal et al., 2004). The L1 capsid protein having conserved sequence is responsible for HPV-host cell interaction for the entry of HPV, accounting to persistent infection in cancer cells and has shown binding affinity in HeLa cell line (Vera-Bravo et al., 2003). The hyper-methylation of L1 gene was highly correlated with the severity of neoplasia in HPV 18 positive clinical samples (Turan et al., 2007) and it affected the transcriptional expression of other genes in HeLa cell line (Badal et al., 2004). The aim of this study is to determine the viral load in HeLa cell line after treatment with the extract of fig fruit to find its anti-viral effect. The viral load is determined widely, by testing the presence of viral capsid proteins and is responsible for virion maturation, assembly, package and release. Many studies have tested the gene for capsid protein L1 to determine the viral load of HPV (Hart et al., 2001) and the L1 ORF has type-specific conserved domain of its protein. The consensus of PCR primers for the highly conserved region in L1 ORF was used for HPV identification (Qu et al., 1997). Hence, we studied the viral load by following the conventional method of detecting the L1 gene in HeLa cell line. In this study, we have utilized the widely used and clinically evaluated MY09/MY11 consensus primers (Strand et al., 2009; Perrons et al., 2002), which amplify the L1 region of HPV in HeLa cells (Burd, 2003).

The currently used therapeutic agents for treating cervical cancer such as; Cisplatin, Paclitaxel are cytotoxic in nature. But the persistence of high risk HPV 16/18 infection keeps transforming the cervical epithelial cells. Hence, there is a need for new drugs, which can reduce the HPV viral load and thereby, preventing cervical cancer development. Traditionally, several medicinal plant sources have been used to treat viral infections. There has been a history of treating cancer and HPV associated infection by using medicinal plants (Park and Pezzuto, 2002; Mori et al., 1998). 

Among these, Ficus is one of the plant species found to be used as both food and medicine. The aqueous extract of the bark of Ficus religiosa has been reported for its apoptosis induction in HeLa cell lines and it has reduced the HPV 18 onco-proteins E6 and E7 (Choudhari et al., 2013). In Iranian medicine, the latex of fig fruit is used to treat papillomatosis, hypoglycemic induction, cancer suppression and anti-helminthic effect (Mostafaie et al., 2011). The ‘Indian Fig’ fruits (Ficus benghalensis L.) belonging to the Ficus family are widely available. Ficus benghalensis L. species have antioxidant property with singlet oxygen quencher molecule. The methanol extract of the bark of Ficus benghalensis L. possess hepato-protective activity in rats (Baheti and Ramesh, 2011). An effective cytotoxic agent namely 6-O-acyl-d-glucosyl-sitosterols has been isolated from fig fruit latex and found to show in vitro inhibitory effects on proliferation of various cancer cell lines (Ghadam et al., 2011). It has been found that Ficus benghalensis L. fruit extract has various effects on the cancer cell line, which includes cytotoxic activity and apoptosis (Ramzan, 2015). Hence, we have evaluated the anti-cancer effect of Ficus benghalensis L. fruit extract on HPV 18 containing cervical cancer cell line (HeLa) in vitro. Furthermore, its antiviral effect on HPV 18 gene upon treatment was analysed in this study by assessing the viral load of HPV 18 in treated cell lines.

## Materials and Methods

*Sample Extraction*

The Ficus benghalensis L. fruits were collected and authenticated by the Botanical Survey of India (BSI). The authentication number is BSL/SRC/5/23/2017/Tech./2923 and the letter is kept for future reference. The fruits were washed and dried at room temperature and by using methanol as solvent, the majority of the compounds were extracted by using soxhlet apparatus. The obtained extract appeared as semi-solid brown paste and stored in a refrigerator until use.

*HPV18 containing cervical cancer cells*

HeLa cells were purchased from NCCS Pune, India and were grown by following the instructions using RPMI1640 (Sigma, UK) as medium. Each 500 ml of the medium consisted of 5.2 g of RPMI powder, 1 g of sodium bicarbonate, 5 ml of sodium pyruvate (1 mM), 5 ml of L- glutamine (2 mM) and 50 mL of heat inactivated foetal bovine serum (FBS) along with Streptomycin (Sigma, UK) in deionised water. The medium was then sterilised by filtering through 0.22 μM pore filter. The pH of the medium was adjusted to 7.4 using concentrated HCL or NaOH and kept at 4°C before use. Cell lines were maintained in a humidified atmosphere of 5% CO_2_ at 37°C. 

*MTT Assay*

The inhibitory activity of the methanolic extract of Ficus benghalensis L. towards HeLa cell line was determined by using the colorimetric method of tetrazolium salt; 3-(4,5-dimethylthiazol-2-yl) 2,5-diphenyltetrazolium bromide (MTT) reduction (Van et al., 2011). Briefly, HeLa cells suspension with 80-90% confluence was added in triplicates into 96 well cultured plates at concentrations of 1x10^5^ cells. After 24 hours of incubation, the fruit extract was added at different concentration (50 µg, 100 µg, 150 µg, 200 µg, 250 µg, 300 µg, 350 µg) and the DMSO was added for control. Then, 20 μL of MTT (5 mg/mL) was added to all the wells after 24 hours. The reduction of yellow MTT into insoluble dark blue coloured formazan product was measured. The insoluble dark formazan products were then solubilized with an organic solvent (DMSO) and the solubilized formazan were then measured spectrophotometrically at 570 nm using a micro ELISA plate reader and the percentage of cell viability was calculated using the following formula:

Percentage cell viability = Absorbance of control - Absorbance of test X 100

*Absorbance of control*

*DNA Fragmentation Assessment*

DNA extraction was performed from 1x10^5^ cells of the cell suspension for both treated (211.86 μg/ml (IC_50_)) and untreated cells. After treatment, cells were centrifuged at 3,000 rpm for five min and the pellet was suspended in 300 μL of cell lysis solution, containing SDS 0.5% and proteinase K to a final concentration of 0.05 ng/mL and incubated at 56°C, overnight. After incubation, equal volume of phenol: chloroform: isoamyl alcohol was added and the aqueous layer was separated. The DNA was then precipitated by adding ammonium acetate (1.4 mM) and an equal volume of ice cold isopropanol. It was then centrifuged at 10,000 rpm for 15 min at 4°C and the obtained DNA pellet was washed in ice-cold 95% ethanol, and again centrifuged at 14,000 rpm for 10 min at 4°C. The pellet was dried and suspended in 100 μL of TAE buffer. This DNA sample was loaded in 0.8% agarose gel, and the DNA bands were observed and analysed using gel documentation system (Bakshi et al., 2010).

*UV Spectrophotometric Analysis*

In order to check the hyper chromic effect of the DNA sample as an indication of DNA damage, the absorbance of the treated (211.86 µg/ml) and untreated DNA sample at 260 nm were noted. Milli Q water was used as blank (Teare et al., 1997). 

*PCR Analysis for HPV 18 Gene:*

The isolated DNA was analysed for the concentration, purity and integrity by measuring them at wavelength A260 and A280 as well as by performing 0.8% agarose gel electrophoresis. The DNA samples having the ratio of A260/A280 as 1.8-2 and a thick band near the well in agarose gel were quantified and subjected to PCR. The DNA of control cell lines was not intact in agarose gel. The isolated DNA samples of both treated and untreated cells were amplified through PCR amplification, which target a portion of the HPV LI region (approximately 450 bp). The amplification of ß-globin fragment (268 bp) was carried out as an internal control to indicate that the sample was adequate for HPV analysis and that no inhibitor was present. Each 25 μL reaction mix contained 12.5 μl Taq DNA polymerase 2x Master mix, 1 μl of forward and reverse HPV LI consensus primers (Depuydt et al., 2007) (MY09 and MY11) (Table 1), and 1 μlof DNA sample, made up to 25 μl using mili Q water. The PCR conditions were set for 40 cycles with denaturation at 95°C for 1 min, annealing at 55°C for 1 min, extension at 72°C for 2 mins and final extension of 7 mins at 72°C. Five microliters of each completed reaction, was electrophoresed on a 2% agarose gel, stained with ethidium bromide and visualized using gel documentation system (Sibele et al., 2016). The band density in the gel was quantified by GelQuant.Net software.

## Results

*Cytotoxicity by MTT Assay*

**Figure 1:**
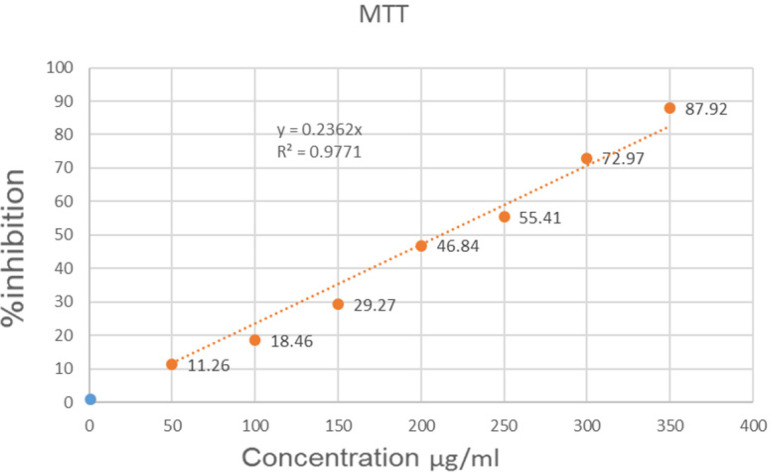
Linear Graph between % Inhibition of Cells and Various Concentration of Fig Fruit Extract

The cytotoxic effect of methanolic fig fruit extract in terms of % inhibition was found by carrying out MTT assay (Table 2). The approximate concentrations of the extracts required, to reduce viability of the cells to about 50% (IC_50_) was found to be 211.86 μg/ml in the given graph (Figure 1). A maximum of 87% inhibition was obtained on cell growth at a concentration of 350 μg/ml of fig fruit extract. 

*DNA Fragmentation*

**Figure 2:**
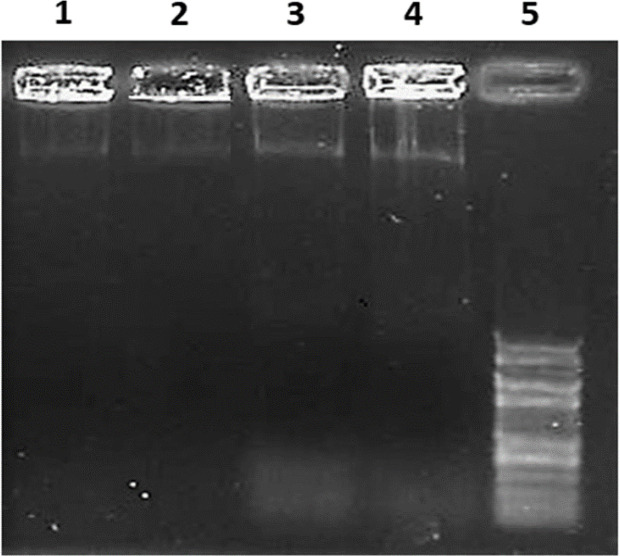
DNA Fragmentation in Agarose Gel Electrophoresis: 1. Untreated 24 hrs sample (control) showing intact DNA near the well; 2: Untreated 48 hrs sample (control) showing intact DNA near the well; 3: DNA cells treated with 211.86 μg/ml (IC 50) for 24 hrs, showing fragmentation. 4: DNA of cells treated with 211.86 μg/ml (IC 50) for 48 hrs, showing extensive fragmentation. 5: 100 bp DNA marker

Cleavages of DNA at inter nucleosomal linker sites yielding DNA fragments are regarded as a biochemical hallmark of apoptosis. In our study, DNA fragmentation was noted in HeLa cells at a concentration of 211.86 μg/ml for 24 hrs of treatment and more fragmentation for 48 hrs treatment. The control cells showed only intact DNA rather fragmentation when subjected to agarose gel electrophoresis (Figure 2).

*DNA Damage Assessment by Hyper-chromic effect Analysis*

The UV Spectrophotometric readings at 260 nm indicated that the DNA sample treated with fruit extract has resulted in hypochromic effect as an indication of increased DNA fragmentation. This resulted in absorbance level of Ficus benghalensis L. fruit extract treated samples compared to that of the untreated DNA sample (Table 3).

*HPV Viral Load Analysis Using PCR*

**Figure 3:**
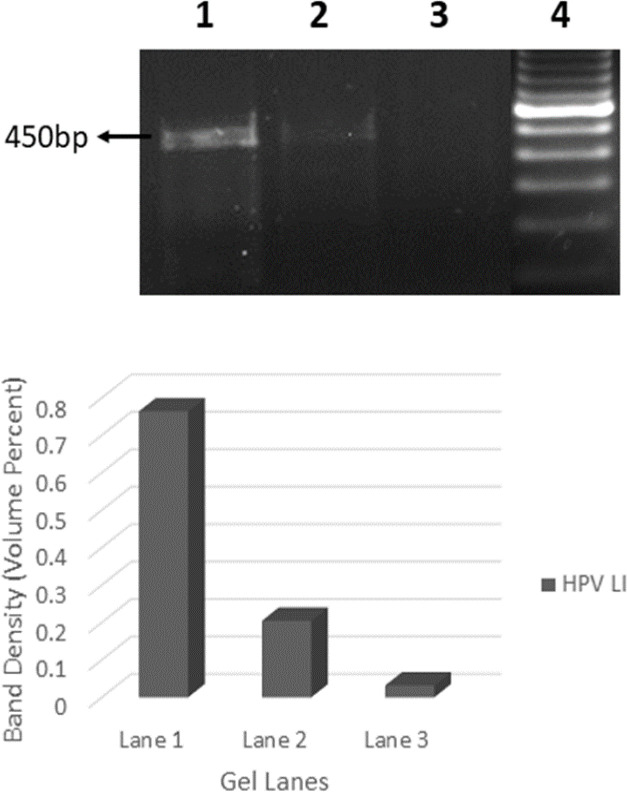
A. Expression of HPV LI gene: 1. Untreated cell line group showing full expression of HPV gene; 2. Cell line treated with Ficus benghalensis L. fruit extract at 211.86 μg/ml for 24 hrs showed very less expression of HPV 18. A light band was observed in 450 bp region in the gel. 3. Sample treated with Ficus benghalensis L. Fruit extract at 211.86 μg/ml for 48 hrs showed no expression of HPV 18; 4. 100 bp DNA marker. B. The band density of HPV LI gene expression for different experimental groups

Successful amplification of the HPV18 L1 fragment is indicated by the 450 bp PCR product band in 2% agarose gel for the untreated sample (Figure 3.A). The PCR product of the sample treated with Fig fruit extract for 24 hrs showed very less expression of HPV 18, and the sample treated with fig fruit extract for 48 hrs showed almost no expression of HPV. The band density of the amplified HPV18 DNA is given in Figure 3.B and the internal control β-actin DNA amplification is shown in Figure 3.C.

**Table 1:** Primer Sequence Used in the PCR Amplification

Name	Sequence	Target	Purpose
MY09	CGTCCMARRGGAWACTGATC	HPVL1	PCR consensus primer (-)
MY11	GCMCAGGGWCATAAYAATGG	HPVL1	PCR consensus primer (+)
PC03	ACACAACTGTGTTCACTAGC	ß-globin	PCR primer (+)
PC04	CAACTTCATCCACGTTCACC	ß-globin	PCR primer (-)

**Table 2:** The HeLa Cells Treated with Fig Fruit Extract at Different Concentration and Their Inhibition. The IC_50 _value of the Ficus benghalensis L. against Hela cell was 211.86

S. No	Concentration (μg/ml)	Inhibition (%) of cell growth
1	50	11.26
2	100	18.46
3	150	29.27
4	200	46.84
5	250	55.41
6	300	72.97
7	350	87.92

**Table 3:** The Values Represented as mean±SD. *P<0.05, **P<0.01, ***P<0.001. Spectrophotometric readings of the DNA samples at different timings (Untreated and treated) DNA samples treated with Ficus benghalensis L. fruit extract has resulted in hypochromic effect. When the untreated cells compared with the treated group at two different durations (24 and 48 hrs), showed a significant difference i.e P<0.01 (**).

Sample	Absorbance at 260 nm (OD)
Untreated cells at 24 hrs	0.04±0.02
Untreated cells at 48 hrs	0.06±0.02
Treated with 211.87 μg/ml fig fruit extract for 24 hrs	0.95±0.07 **^a^
Treated with 211.87 μg/ml fig fruit extract for 48 hrs	1.01±0.03**^b^

## Discussion

Cervical cancer is the second leading cancer among women with an estimation of 445,000 new cases in 2012 (84% of the new cases worldwide). According to WHO (World Health Organisation), more than 85% of deaths occurs in low and middle income countries. It is stated that about 70% of cervical cancers occurs in developing countries. HPV infection is the major cause of cervical cancer, but finding the HPV treatment effect at molecular level is a difficult task. The HPV18 inclusions and L1 capsid proteins were observed in HeLa cell line which suggest the presence of L1 gene in HeLa cells and its ability to express L1 proteins (Xiao et al., 2015). In order to develop a novel approach in the treatment of cervical cancer to target the HPV, we have used the Ficus benghalensis L. plant material as the plant materials have been used traditionally for HPV treatment as therapeutic agent (Lansky et al., 2008). The current study was thus, focused on finding the effect of Ficus benghalensis L. fruit extract against the HPV generated from the integrated genome in the cervical cancer cells. The cytotoxic activity of Ficus benghalensis L. fruit extract against HeLa cell line was analysed in this study by MTT assay. Ficus ariculata was earlier shown to possess anticancer activity against HeLa cell line, indicating an IC_50_ value of 1300 μg/ml at 24 hrs of incubation (Ritu et al., 2012). Our present study on Ficus benghalensis L. showed a higher cytotoxic activity against HeLa cell line, with an IC_50_ value of 211.86 μg/ml at 24 hrs of incubation. 

The DNA fragmentation analysis is done for understanding the apoptotic activity and deactivation pattern of the gene codes, for the enzymes involved in cancer. The DNA of apoptotic cells undergoes fragmentation as shown in many studies (Roos and Kaina, 2006). The Apoptosis (cell death) and its signalling pathways have a remarkable impact on the progression of cancer (Lowe and Lin, 2000) and initiation of apoptosis is a highly desirable goal for controlling the cancer and its pathways (Reed and Pellacchia, 2005). In our study, the DNA fragmentation was observed in HeLa cells treated with Ficus benghalensis L. fruit extract at a concentration of about 211.86 μg/ml. The control cancer cells did not show any fragmentation as shown in Figure 2. The UV absorption at 260 nm will be high when the DNA strands are being separated, either by heat or by addition of denaturant or by increasing the pH level (Wang et al., 2014) by hyperchromic effect. The DNA fragmentation due to apoptosis were also reported to increase the absorption level and analysed by UV spectrophotometry (Teare et al., 1997). In the present study, it was observed that the DNA sample of HeLa cells treated with Ficus benghalensis L. fruit extract showed OD values higher than the DNA sample from the untreated HeLa cells. These results imply that the Ficus benghalensis L. fruit extract could induce the apoptosis associated DNA fragmentation in HeLa cell lines at 211.86 µg/ml concentrations.

Analysing the presence of Human Papilloma Virus (HPV), its types and its viral load is the basic step in evaluating the risk of cervical cancer and most of the available data indicate that the cervical lesions are related to HPV-16 and HPV-18 (Clifford et al., 2006). The HeLa cell line was used as positive control for the presence of HPV 18 in many studies (Peran et al., 2010; Xiao et al., 2015). The latex of fig fruit and other parts were reported to be traditionally used for HPV treatment. In this study, we intend to analyse the effect of Ficus benghalensis L. fruit extract on the HPV viral load in HeLa cell lines by PCR method. In the current investigation, the difference in HPV 18 viral load between the treated and untreated HeLa cells was evaluated using PCR method by treating the cells with 211.86 μg/ml (IC_50_). Majority of HPV studies have used the MY09/11 primers that are potentially capable of detecting many mucosal HPV types in a single PCR reaction (Molijn et al, 2005) and are also specific to a highly conserved HPV L1 gene region (Nobre et al., 2008). 

According to Poljak et al., and Vince et al., the chances of false negative result for HPV in PCR detection due to poor clinical sample having low concentration of HPV DNA or by PCR endogenous inhibitors or from sample processing should be evaluated. So, we used human β globin as control in our study and successful amplification of the ß-globin fragment (268 bp) indicated that the sample was adequate for HPV analysis and that no inhibitor was present. Our results have shown a significant reduction in the level of HPV DNA in terms of the band width in agarose gel and the density in treated sample when compared to the control sample as shown in Figure 3. This implies that the Ficus benghalensis L. fruit extract could reduce the HPV load in HeLa cells. However, the molecular mechanism and its effect on HPV gene expressions need to be explored further to validate its use as therapeutic agent for HPV infected cervical cancer. Recent studies reveal that various natural compounds like, curcumin were found to reduce the E6 and E7 mRNA expression of HPV 18 (Yew et al., 2011). From the above results and discussion, it is noted that the methanolic extract of Ficus benghalensis L. fruit has a positive effect on reducing the HPV 18 viral load in HeLa cell lines and in future it can be used as therapeutic drug for cervical cancer.

In conclusion, this study illustrates the decrease in HPV 18 viral load upon treatment with methanolic Ficus benghalensis L. fruit extract as compared with the untreated HeLa cell line. The cytotoxic activity of fig fruit extract on Hela cells also showed a high percentage inhibition of about 87.92% for 350 μg/ml for an incubation period of 24 hrs. The enhanced DNA fragmentation observed in treated cell lines in terms of DNA laddering and hyper chromic effect implies that the apoptotic activity was induced by the effect of Fig fruit extract treatment.

## Author Contribution Statement

Conceptualization: V. M. Berlin Grace. Investigation and Data curation: B. Lydia. Methodology: V. M. Berlin Grace. Project administration: D. David Wilson. Writing – original draft: V. M. Berlin Grace and D. David Wilson. Review & editing of manuscript: D. David Wilson

## Approved by any scientific Body

Approved by the internal Project Review Panel of the Department of Biotechnoloy, Karunya Institute of Technology and Sciences

## Part of an approved thesis

No.
